# Intraoperative Radiation Therapy for Gynecologic Malignancies: When Is It Indicated?

**DOI:** 10.3390/cancers17071240

**Published:** 2025-04-06

**Authors:** Flavia Pagano, Flurina Annacarina Maria Saner, Codruta Ionescu, Elena Riggenbach, Kristina Lössl, Franziska Siegenthaler, Michael David Mueller, Sara Imboden

**Affiliations:** 1 Department of Obstetrics and Gynecology, University Hospital of Berne, University of Bern, 3010 Bern, Switzerland; flavia.pagano@insel.ch (F.P.); flurina.saner@insel.ch (F.A.M.S.); michel.mueller@insel.ch (M.D.M.); sara.imboden@insel.ch (S.I.); 2 Department of Radiation Oncology, University Hospital of Berne, University of Bern, 3010 Bern, Switzerland; codruta.ionescu@insel.ch (C.I.); elena.riggenbach@insel.ch (E.R.); kristina.loessl@insel.ch (K.L.)

**Keywords:** cervical cancer, complication rates, ECOG performance status, endometrial cancer, Intraoperative radiation therapy, pelvic malignancies

## Abstract

**Background**: Intraoperative radiation therapy (IORT) is the delivery of a single large dose of radiation to a limited volume of tissue at the time of surgery. The aim of this study is to assess outcomes in patients who underwent IORT for gynecologic malignancies and to identify parameters that can predict a good outcome. **Methods**: This is a retrospective single-center cohort study including all women treated with surgery and IORT for a primary or recurrent gynecologic cancer between 2014 and 2022 at the Bern University Hospital, Switzerland. **Results**: A total of 30 patients with gynecologic malignancies were treated with surgery and IORT. Of these patients, 63.3% presented with cervical cancer, 23.3% with sarcoma, 10% with endometrial cancer, and 3.3% with carcinosarcoma of the ovary. Seventy percent (21/30) of women had an ECOG performance status of 0 at time of IORT. There was no difference in survival among women with incomplete surgical resection (R1/2 vs. R0) at time of IORT. Fifty percent of patients suffered postoperative complication of Clavien-Dindo grade >III, but there was no significant correlation of these complications to overall survival (*p* = 0.58). Three-year disease-free survival was 53.3%, and five-year overall survival was 53.3%. ECOG status was a significant parameter in DSS (*p* = 0.002) and OS (*p* = 0.02). **Conclusion**: Surgery with IORT is potentially a good treatment option in selected patients with recurrent or locally advanced cervical or endometrial cancer. An ECOG status of 0 is a significant parameter for good outcomes and should be taken into consideration for treatment decisions.

## Simple Summary

This retrospective study shows that patients with locally advanced or locally recurrent cervical or endometrial cancer may benefit from surgery combined with intraoperative radiation therapy. An Eastern Cooperative Oncology Group (ECOG) status of 0 is essential for a good outcome. These treatments show a high morbidity. However, there is no significant correlation between these complications and overall survival.

## 1. Introduction

About 94,000 women worldwide are diagnosed with gynecologic cancer each year; over 28,000 women succumb to their disease annually. Primary treatment in most cases involves surgery and/or external beam radiotherapy (EBRT), since most gynecologic malignancies are radiosensitive. The management of recurrent gynecologic cancers remains a challenge and is mostly multimodal. Chemotherapy alone is usually indicated only in a palliative setting. Surgery is a treatment option in patients with loco-regional cancer recurrence and is often followed by postoperative radiotherapy.

However, particularly in women with cervical and endometrial cancer, patients with tumor recurrence often have a history of previous irradiation as either first-line primary treatment (mostly for cervical cancer) or adjuvant treatment (mostly for endometrial cancer).

Therefore, re-irradiation for relapsed disease with the aim of controlling the disease without damage to the surrounding tissue is often not feasible. Moreover, microscopic residual disease that remains in patients undergoing salvage surgery for recurrent gynecologic malignancy is difficult to treat with EBRT because of its localization in the pelvis with close organs such as the intestine, bladder, and ureter, and because of recurrence often lying within a previously irradiated field [1].

Intraoperative radiation therapy (IORT) is a technique designed to deliver a single large dose of radiation to a focused region at the time of surgery [2,3]. IORT has been used in primary management as well as in a salvage setting for many solid tumors [4] and is a unique modality that facilitates the treatment of microscopic disease [1]. With IORT, the healthy organs and tissue can be moved away from the treatment field during radiotherapy [1,5] Therefore, for a single fraction, high dose radiation can be delivered with minimal toxicity risk to surrounding tissues [1,6].

It is known that the biological effect of this single massive dose is equivalent to 2–3 times the dose administered by conventionally fractionated treatment [4]. Another advantage of IORT is that it is administered immediately after tumor resection, allowing direct visualization of the target area, with collaboration between the radio-oncologist and surgeon [4].

IORT can be delivered via two techniques: the electron beam technique and HDR brachytherapy. In the electron beam technique, radiation is delivered by a linear accelerator and directed to the tumor bed with a cone [6,7]. In HDR brachytherapy, catheters within a 1 cm thick tissue-equivalent material are placed along the tumor bed and loaded with iridium-192 [1,2]. Electrons generated by LINACs and brachytherapy sources can be conveniently used for IORT procedures in gynecologic and genitourinary tumors [2].

Several studies have shown that the application of IORT in patients undergoing surgery for recurrent gynecologic cancers can result in improved local disease control and improved long-term overall survival [1,7,8,9]. However, IORT is still used primarily for selected cases with individual indications. The aim of this study is to analyze the cases of patients who underwent IORT for gynecologic malignancies and to identify parameters that can predict a good outcome.

## 2. Materials and Methods

In this single-center cohort study, all patients treated with surgery and IORT for a primary or recurrent gynecologic cancer at the University Hospital of Bern, Switzerland, between January 2014 and December 2022 were included. Upon approval of the local ethics committee (reference number: 2018-00479), data from all patients undergoing IORT in this Swiss tertiary referral center were collected from the hospital internal patient database and the prospectively collected database on oncological tumors for follow-up (ODSeasy, Version 5.5.0.0, Asthenis medical GmbH, Aschheim, Germany). We analyzed patient, tumor, and peri-and postoperative characteristics, as well as follow-up data on oncological outcome.

Preoperatively, a complete blood count, blood chemistry tests, and computed tomography or positron emission tomography (in function of the physician’s preference) were performed for each patient. Gross total resection with macroscopically negative margins was attempted in every case. Indications for IORT involved cases where surgery alone would not provide acceptable local control, and EBRT was not an option due to either anatomical constraints, comorbidities, or previous radiotherapy to this area with exhausted dose.

The IORT procedure was performed using high-dose-rate (HDR) brachytherapy with an Iridium-192 stepping source. A silicone-based flap applicator was used that can be cut to the required size and conforms to curved anatomical surfaces. This flexible, mesh-style surface mold consists of silicone rubber beads. An array of catheters, spaced 10 mm apart, is embedded in parallel, while a consistent source-to-tissue distance of 5 mm is maintained. The applicator was positioned under direct visualization of the tumor bed, targeting areas deemed to have the highest risk of residual tumor or where margins were anticipated to be close. A dose of 1000 cGy was prescribed at 0.5 cm tissue depth from the surface of the applicator, resulting in an approximate surface dose of 1500–1600 cGy. The same dose was used for all patients, independent of previous irradiation. Uninvolved ureters, nerves, and vascular structures were excluded from the IORT area or distanced from it whenever possible. A maximum point dose constraint of 1700 cGy at the applicator surface was applied to limit IORT-associated toxicities.

Overall survival (OS) was defined as time from primary diagnosis until death due to any cause or last follow-up. Disease-specific survival (DSS) is the length of time from the start of treatment for a disease, such as cancer, to the date of death from the disease. Patients who die from causes unrelated to the disease are not counted in this measurement. Relapse-free survival (RFS) is based on the time from after primary treatment for a cancer ends that the patient survives without any signs or symptoms of cancer to the time of recurrence or death. In patients who had recurrent disease, the previous recurrence-free interval was evaluated. After IORT treatment, we measured time intervals from day of IORT to most recent follow-up visit, or disease recurrence or cancer-related death. Local failures were defined as disease recurrence in the IORT field. Regional failure was defined as recurrence within the abdomen and pelvis, but outside the IORT field. Distant failure was defined as disease relapse outside the abdomen and pelvis. Follow-up information was available for all patients from our oncological follow-up database for certification processes (ODSeasy).

Intraoperative complications were classified using CLASSIC (Definition and Classification of Intraoperative Complications) [10], and postoperative complications using Clavien-Dindo classification [11].

Statistical analysis was performed using SPSS Version 25.0 (IBM^®,^, Armonk, NY, USA). Demographic and clinical pathologic characteristics were evaluated using basic descriptive statistics, with comparison of the groups using the chi-square test and the Mann–Whitney *t*-test.

All *p*-values were two sided, and *p*-values < 0.05 were considered to be statistically significant. The Kaplan–Meier method as well as Cox regression analysis were used to evaluate the oncological outcome.

## 3. Results

### 3.1. Patient and Tumor Characteristics

From January 2014 to December 2022, we treated 30 patients with gynecologic malignancies with surgery and IORT. Four other patients were planned for IORT, but the procedure could not be performed due to positive tumor margins (R2 resection). The demographic data and tumor characteristics of the 30 patients are summarized in Table 1.

**Figure 1:**
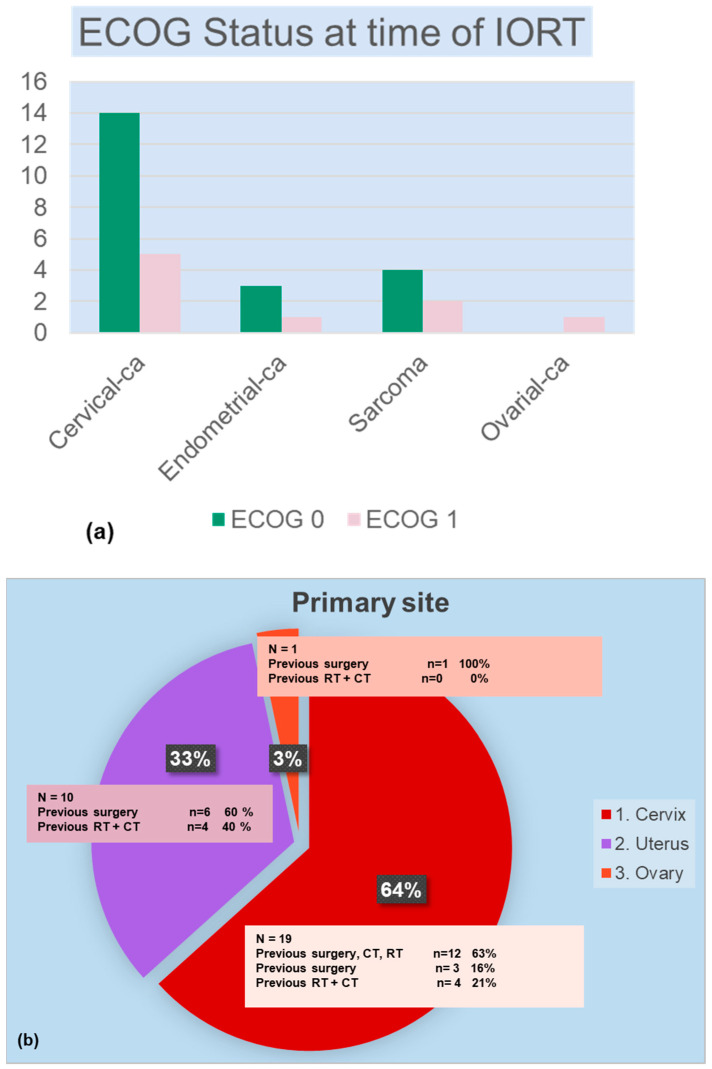
ECOG status at time of IORT (**a**), primary site (**b**).

Patients were treated with IORT for four different pelvic malignancies: nineteen (63.3%) for cervical cancer, three (10%) for endometrial cancer (two endometrioid and one clear cell adenocarcinoma), seven (23.3%) for uterine sarcoma, and one (3.3%) for a carcinosarcoma of the ovary. At initial diagnosis, 20 patients had a FIGO stage I/II cancer (66.6%) and 10 (33.3%) had advanced stage (III/IV) disease. Twenty-four patients (80%) received IORT for recurrent disease. Figure 1a shows the ECOG status at the time of IORT, and Figure 1b shows the primary sites of tumors that were treated and the main patient characteristics. The majority had an ECOG Status of 0 and an advanced FIGO stage of III/IV (n = 26, 86.7%, mainly IVa 62.3%) at the time of IORT. The median time between the end of primary treatment and IORT in patients with recurrent disease was 12 months (range 1 to 312 months), and three (10%) patients had no pretreatments before IORT (surgery, adjuvant therapy). In Table 2, we describe patients’ treatment history before and at IORT. Most patients had a prior surgery (n = 26, 86.6%) and radiotherapy (n = 21, 70%). A total of 63.3% of patients had been treated with a systemic therapy before IORT; regimens included primarily cisplatin and 5-fluoracil.

### 3.2. Surgical Data

In all patients, the goal of the intervention was the complete surgical resection of the tumor/recurrence and the performance of local IORT. To achieve this goal, 19 (63.3%) patients had a hysterectomy, 13 (43.3%) needed bowel resection, 21 (70%) had radical lymph node dissection, and 9 (30%) needed ureteral resection, and of these, 7 (23.3%) required a cystectomy. Surgical data are described in Table 2. Regarding the specific type of surgery, 11/30 (36.7%) patients underwent pelvic surgery, 3/30 (10%) patients underwent extended lateral pelvic exenteration to the pelvic wall, 7/30 (23.3%) patients had abdominal tumor resection, and 9/30 (30%) patients had tumor resection with radical pelvic and/or paraaortic lymph node dissection.

In nine patients (30%), surgical resection margins were microscopically positive based on frozen section. One patient with minimal disease (<1 cm) who was not macroscopic tumor free was also included, when no other options were available.

This patient with uterine sarcoma underwent laparotomy with hysterectomy, bilateral adnexectomy, and radical pelvic and paraaortic lymphadenectomy. A biopsy from the posterior vaginal wall was sent for frozen section analysis, as well as from the right pararectal tissue and right pelvic wall, which showed infiltration by carcinoma. After further removal of tissue from the right pararectal area and right pelvic wall, the pelvis appeared macroscopically tumor-free. However, based on these frozen section results, it must be considered an R1 resection. To achieve an R0 resection, a total pelvic exenteration would be required. However, given the biological nature of carcinosarcoma, this is not indicated due to the increased risk of distant metastases and IORT was performed in sites of positive frozen sections.

All 30 patients were treated with IORT. The mean dose delivered was 1000 cGy, with a mean of 8 applicators/seeds (range 2–16). In three patients (10%), IORT was used to treat the pelvic sidewall. The presacral nodal region and central pelvis were treated in two (6.6%) and twenty-one (70%) patients, respectively (Table 2). After IORT, fifteen patients (50%) received adjuvant therapy: three (10%) received EBRT, five (16.6%) received chemotherapy, five (16.6%) received EBRT and chemotherapy, and two (6.6%) received anti-hormonal therapy (GnRh analogue treatment) for recurrent uterine sarcoma.

The chemotherapy regime used was carboplatin and paclitaxel-based.

Complications during surgery and after IORT are summarized in Table 3.

In eight (26.7%) patients, intraoperative complications were noted: in one patient, a CLASSIC Grade 3 complication occurred (bleeding from the presacral venous plexus and consequent lesion of the ischiadic nerve). Half of the patients had Clavien-Dindo Grade III or IV complications postoperatively, showing the high morbidity of these surgeries when combined with IORT. A search for risk factors for these complications revealed that only pre-surgical pelvic radiation was a significant risk factor (*p* = 0.02, Table 4).

We also analyzed the long-term complications, such as persistent symptoms due to the surgery. Of the patients alive at time of analysis, four patients (13.3%) had persistent or recurrent problems due to surgery: one with ureteral stricture needing reconstruction, one with recurrent pyelonephritis due to cutaneous ureterostomy with stents, one with an ischiadicus lesion, and one with recurrent abscesses around the colostoma. No clear significant risk factors could be identified.

The median length of hospital stay after surgery and IORT was 13 days (range 3–100 days).

One patient remained in the hospital for 100 days due to complications following a pelvic exenteration for recurrent endometrial cancer. Postoperatively, a relaparotomy was required for a small bowel perforation and vaginal stump dehiscence. Additionally, there was a wound healing disorder at the laparotomy incision.

### 3.3. Oncological Outcome

The median follow-up time (IORT to follow-up or death) was 31.8 months (3 to 103 months). Fifteen patients (50%) developed a recurrence after IORT after a median of 7 months (range 3–60 months).

Ten patients (33.3%) experienced a local recurrence in the field of IORT and five (16.7%) developed distant metastatic disease after IORT (Table 2). Treatments of recurrence after IORT included chemotherapy, surgery, immunotherapy, and a second IORT (Table 2). Fourteen patients (46.7%) had died by the time of analysis, of which eleven (36.7%) died due to the disease. OS at 5 years was 53.3% for the entire group, and the 1-year RFS rate was 70%.

We analyzed parameters that could have had an impact on recurrence and survival; these are summarized in Table 5.

**Figure 2:**
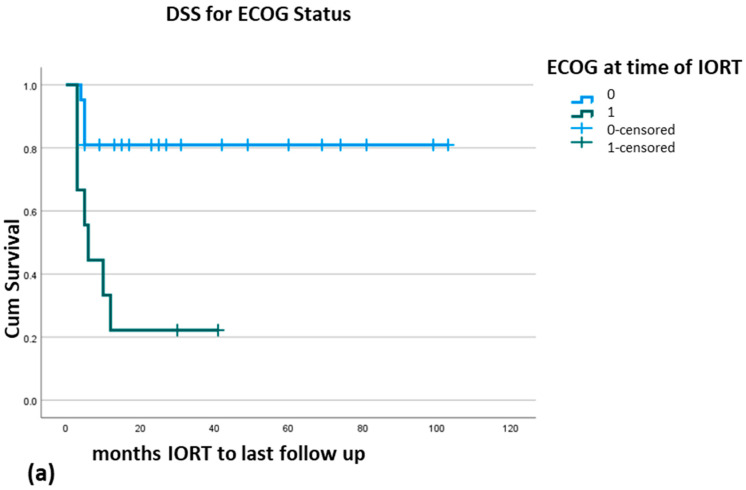

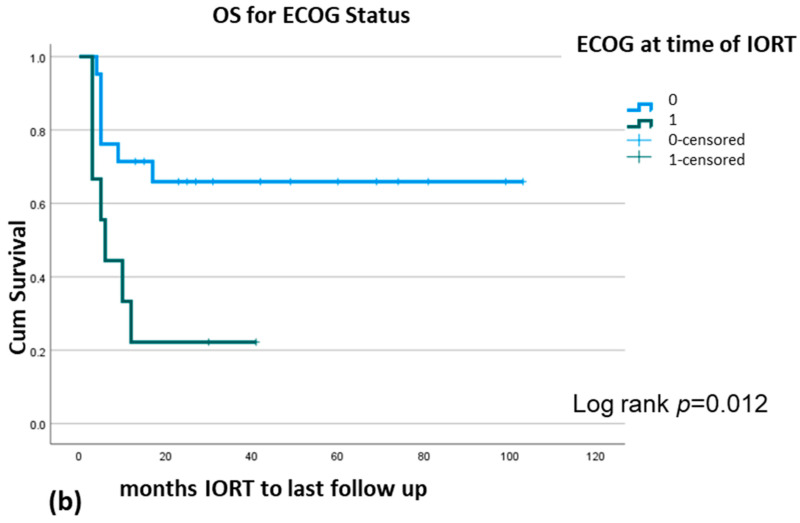
Disease specific survival = DSS for ECOG status (**a**), overall survival = OS for ECOG status (**b**).

**Figure 3:**
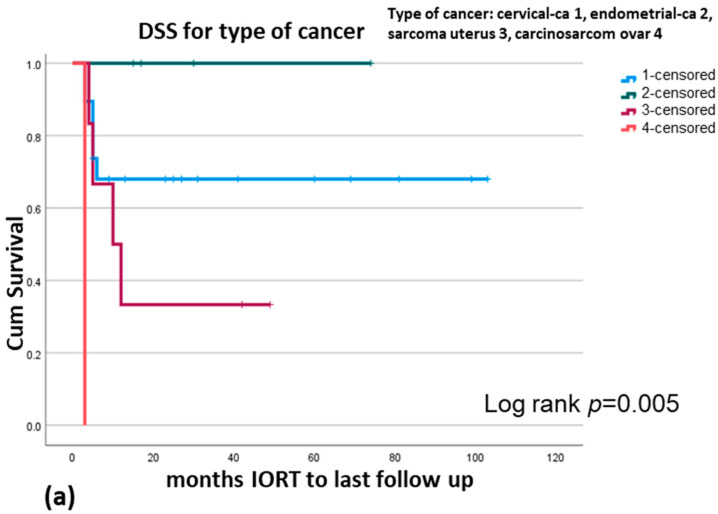

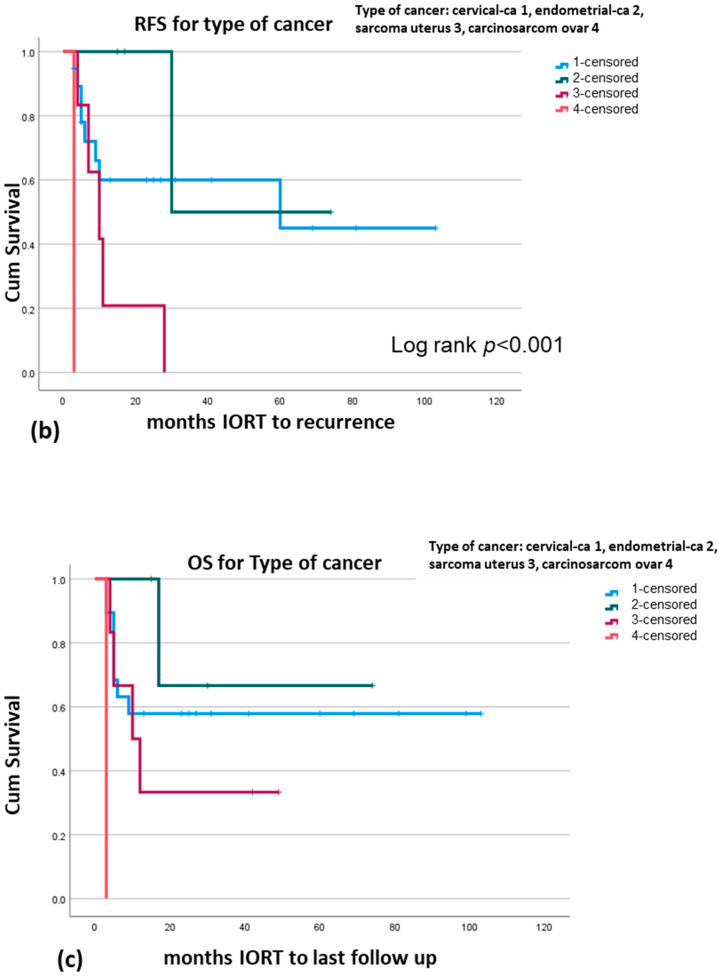
Disease specific survival = DSS for type of cancer (**a**), relapse-free survival = RFS for type of cancer (**b**), overall survival = OS for type of cancer (**c**).

Significant findings are the importance of ECOG status in DSS (*p* = 0.002) and OS (*p* = 0.02) (Figure 2a,b) and the type of cancer for DSS (*p* = 0.008), for RFS (*p* ≤ 0.001), and for OS (*p* = 0.02) (Figure 3a–c). In multivariate analysis, ECOG remains a significant parameter for DSS (*p* = 0.02) and HR 4.76 (CI 95% 1.27–17.81) (Table 5). OS is no longer significant at multivariate analysis (*p* = 0.06, HR 3.01 (CI 0.96–9.41)).

## 4. Discussion

This study describes the experience with IORT in the treatment of gynecologic malignancies at the University Hospital in Bern. In this complex cohort, we found that surgery with IORT led to a satisfactory survival rate, with an OS of 53.3% for the entire group at a median follow-up time of 31.8 months. Patients with locally advanced primary or locally recurrent gynecologic cancers have generally poor prognosis and limited treatment options, the latter due to the need for heavy pre-treatments. These patients are shown to possibly benefit from surgery combined with IORT as a therapeutic option [5,7]. The reported 5-year survival for patients with locally advanced gynecologic malignancies requiring pelvic exenteration ranges from 20% to 50% [5]. For patients who had undergone previous irradiation, the doses of EBRT required to achieve adequate tumor control often exceed dosages tolerable by normal tissue [4,5]. In these cases, IORT may offer an adjunctive treatment modality to radical surgery alone. Several studies have shown that IORT improves both long-term control of the disease and the OS of women with pelvic sidewall tumor extension and/or para-aortic nodal recurrences [5,12].

However, these cohorts are always very inhomogeneous and in guidelines, we did not find clear criteria to determine which patients are good candidates for these extensive surgeries. Surgery with IORT is always individual, and it is hard to execute perfect patient selection.

The aim of our study was therefore to detect factors that help identify who can potentially benefit from this form of surgery. We found that patients with cervical and endometrial cancer, as well as ECOG 0, have the best oncological outcome. Consistent with other published series, we also report relatively positive outcomes for a group of patients with a poor prognosis historically, especially in the setting of tumor extension to the pelvic sidewall or lymphatic dissemination to the pelvic or para-aortic lymph nodes [5,7,12,13].

The majority of case series documented in the literature describe a heterogeneous cohort characterized by varying histologies and tumor origins.

In most case series, the criteria used for the selection of women with gynecologic malignancies who may be candidates for treatment with IORT were: (1) evidence of local recurrence with no evidence of distant disease; (2) medical fitness to tolerate major surgery (however, the ECOG status had never been analyzed prior to our study); (3) inability to achieve acceptable local control through surgical intervention alone; and (4) positive microscopic margins if surgery alone was performed [7,12]. Survival in patients with cervical cancer and central recurrences treated with pelvic exenteration is significantly higher (30–50% 5-year survival) than those patients who were inoperable [7]. This indicates that local control may play a crucial role in enhancing survival rates. Salvage treatment of pelvic recurrences after definitive radiation therapy remains a difficult challenge. Currently, there is no established standard treatment protocol for patients with recurrent gynecologic malignancies with microscopic residual regional disease after surgical intervention [7]. Approximately 60% of individuals with cervical or endometrial cancer will die from local failure. Exenterative surgery is effective only for a small percentage of women with recurrent disease confined to the central pelvic region [12].

Mahé et al. described, in a study of 70 patients with recurrent cervical cancer treated with IORT, a median survival time of 11 months and a 21% local control rate after a median follow-up of 15 months. Of the 70 women, 40 had IORT only, while 30 received IORT and EBRT. Survival rates at 1 and 2 years were 47% and 17%, respectively [14].

Del Carmen et al. described a case series of 15 patients with primary tumors from the cervix, endometrium, ovaries, or vagina; their study reported that in the management of recurrence of gynecologic malignancies, the volume of residual disease before IORT may be an important prognostic indicator of disease relapse [15]. In this series, overall survival at 5 years was 25% and 38% for cervical and endometrial cancer, respectively. Yap et al. reported a case series of 22 patients with recurrent ovarian cancer: five-year OS was 22% with a median survival of 26 months from time of IORT [9]. The predominant histologic subtype was papillary serous (9/22) [9]. Intraoperative radiation therapy was given as adjuvant treatment in cases of isolated recurrent disease in which the resection margins were deemed close or microscopically positive on frozen section analysis, and where adjuvant postoperative EBRT doses for local control would exceed those tolerated by normal surrounding organs [9].

Especially for tumors that are not very chemosensitive, such as soft tissue sarcomas, cervical cancer, endometrial cancer, and vulva cancer, margin status is critical [4]. Residual disease after cytoreductive surgery is an important prognostic indicator in the treatment of gynecologic cancers [5]. Surprisingly, in our study, when looking at oncological outcome, resection margins were not a significant factor. This is in contradiction with the literature, which describes that known factors that predict improved survival include negative margins, small central tumor, prolonged interval form radiation (<2 years), and lack of sidewall involvement [4,5,16]. Positive margins at the time of pelvic exenteration decreased the 2-year OS to 10%, compared with 55% in those with negative margins [7,15,17]. Backes et al. found that patients who receive IORT frequently have positive margins and have a shorter survival than patients who require a pelvic exenteration only (without IORT) [1]. Multiple retrospective studies have demonstrated the importance of negative margins for local control, with increased local and distant recurrences as well as decreased OS with positive margins, even if IORT is used [4,18,19]. The fact that in our group, 21/30 patients (70%) had a complete surgical resection may in part explain the generally good outcome. Whether the lack of difference between clean margins and microscopical residual disease was due to the IORT cannot be proven with this study. However, it motivates us to apply IORT also if margins are close with the chance that these patients might still benefit from surgery combined with IORT. In any case, in the group with microscopic positive resection margins (9/30), 56% received adjuvant treatment, compared to 50% of the R0 group. Therefore, these similar outcomes are likely not attributable to differences in the use of adjuvant treatment.

In general, adjuvant treatments after surgery and IORT (postoperative EBRT, dose of postoperative EBRT, and chemotherapy) were not significant in the delivery of better outcome; this is in line with similar findings reported by Foley [5] and Tran [13].

Half of the patients had Clavien-Dindo Grade III or IV complications postoperatively, showing the high morbidity of these surgeries combined with IORT. This is a high number; however, these patients did not have many other treatment options, and this is consistent with what has been reported in the literature for IORT in the treatment of gynecologic malignancies (major complications have been reported in 50–80%) [2,4,20].

In search of risk factors for intraoperative complications, only pre-surgical pelvic radiation (*p* = 0.02) was found to be a significant risk factor.

Fortunately, no correlation was found between severe complications and oncological outcome, and no patient died as a result of the surgery. This is an important point for patient selection and counseling: we must discuss possible severe complications, especially in patients after radiation therapy. Knowing that they do not compromise oncological outcome could also motivate them to tolerate the surgery. These are facts that are also seen in extensive surgeries for recurrent ovarian cancer treatment, including analysis of quality of life after severe complications. Although the study included a different cohort of patients, the findings from the DESKTOP III trial indicated that major surgery with a high complication rate does not impact on quality of life after the healing phase [21].

In our study, we analyzed long-term complications, such as persistent or recurrent symptoms due to the surgery. Of the patients alive at time of analysis, four patients (13.3%) had long-term problems due to surgery: one with ureteral stricture needing reconstruction, one with recurrent pyelonephritis due to uretero-cutaneous-neostomy with stents, one with an ischiadicus nerve lesion, and one with recurrent abscess around the colostoma. Here, probably due to the small sample size, no clear significant risk factor was identified, and unfortunately, we have no data on quality of life.

The adverse events reported in this series are consistent with complication rates of other studies [5,7,12,13]. The two most common IORT-related complications in the treatment of locally advanced pelvic malignancies identified in previous analyses are peripheral neuropathy and ureteral obstruction [9,22].

In our group, no patient died in the early postoperative period from a surgical complication.

A low rate of neuropathy was observed. Other complications following IORT were attributed primarily to radical/exenterative surgery performed in previously irradiated or radically dissected tissue, rather than to the intraoperative irradiation itself.

ECOG is a known score for easily identifying patients that can tolerate treatment. It is often used by oncologists.

In our study, we observed that patients with an ECOG performance status (PS) of 0 had significantly better DSS and OS. ECOG PS is a multifactorial parameter, and its prognostic relevance may reflect not only the biological aggressiveness or advanced stage of the disease, but also the patient’s overall health and comorbidities. The majority of our cohort had FIGO stage III/IV disease and had undergone multiple pre-treatments, including radiotherapy and chemotherapy. To what extent the ECOG score reflects disease burden versus other frailty-related factors is difficult to assess due to the retrospective nature of the study. Nevertheless, we believe this finding represents an important consideration for patient selection. ECOG PS gives us an easily applicable score to apply in indication for surgery with IORT and for counseling patients. These results suggest that indicating surgery with IORT in patients with an ECOG > 0 should be performed with the greatest care.

### Strengths and Limitations

The principal limitation of our study is its retrospective nature, alongside the small number of patients and the use of a heterogenic cohort with different histologies and tumor origins. However, the extensive data on the patients and the complete follow-up constitute, in our opinion, a valuable contribution to a better understanding of this treatment option, for which only limited data are available. Future large-scale multicenter studies would be needed to prove the efficacy and safety of IORT for women undergoing salvage surgical resection of recurrent gynecologic cancers.

## 5. Conclusions

Surgery with IORT is potentially a good treatment option in selected patients with satisfactory survival. This method was associated with an OS at 5 years of 53.3% for the entire treatment group, who had exhausted treatment options, at a median follow up time of 31.8 months. However, the studied cohort was a heterogeneous group of patients, and the sample size was small, as the authors admit. It is therefore difficult to draw conclusions based on this. Our experience suggests that surgery with IORT for patients with locally advanced or locally recurrent cervical or endometrial cancer has the best prognosis. These interventions are associated with a high morbidity; however, the complications have no effect on the oncological outcome. An ECOG status of 0 is a significant parameter in higher survival rates, not only OS but also DSS, and possibly should be used when making treatment decisions.

No parameters were identified that could reduce the complication rates; however, neither severe complications nor positive resection margins have a negative impact on oncological outcome.

The role of IORT in the treatment of advanced primary and recurrent gynecologic cancers remains an individual decision.

## Figures and Tables

**Table 1:** Pre-IORT patient demographics and tumor characteristics.

median age (range)	50 (32–80)
menopausal status	*n* (%)
premenopausal	12 (40)
postmenopausal	19 (63.3)
recurrent disease	24 (80)
primary site	
cervix	19 (63.3)
uterus	10 (33.3)
ovary	1 (3.3)
histology	
sarcoma	7 (23.3)
squamous	15 (50)
adenocarcinoma	6 (20)
clear cell	1 (3.3)
carcinosarcoma	1 (3.3)
FIGO stage at first diagnosis	
I/II	20 (66.6)
III/IV	10 (33.3)

The patients ranged in age from 32 to 80 years (median age, 52.4 y), and 19 patients (63.3%) were postmenopausal.

**Table 2:** Patients’ treatment history before and at IORT.

Tumor and treatment characteristics	N = 30
pretreatment before IORT, *n* (%)	27 (90)
previous surgery, n (%)	26 (86.7)
previous radation therapy, n (%)	21 (70)
EBRT only, n (%)	10 (33.3)
brachytherapy only, n (%)	1 (3.3)
EBRT and brachytherapy, n (%)	11 (36.6)
chemotherapy before IORT, *n* (%)	19 (63.3)
months between primary treatment and IORT, median (range)	38.2 (3–312)
Mean age at IORT, years (range)	56.9 (35–86)
Mean BMI at IORT, kg/m^2^ (range)	23 (15.92–37.9)
ECOG at time of IORT, n (%)	
__0	21 (70)
__1	9 (30)
IORT site, *n* (%)	
pelvic sidewall	2 (6.7)
pelvis	21 (70)
presacral nodal region	2 (6.7)
abdominal	1 (3.3)
vaginal	4 (13.3)
number IORT Applicator, median (range)	8 (2–16)
IORT dose (cGy)	1000
radiotherapy time in seconds mean (range)	977.8 (270–2350)
total time in min	111.6
blood loss (ml)	1043
Op time in min	460.4
resection status/surgical margin status at time of IORT	*n* (%)
R0	21 (70)
R1 gross residual disease	1 (3.3)
tumor microscopic to the resection margin	9 (30)
FIGO Stage at IORT	
I/II	4 (13.3)
III/IV	26 (86.7)
hospitalisation days median (range)	13 (3–100)
Survival status	
alive	16 (53.3)
Adjuvant treatment after IORT	16 (53)
EBRT	3 (10)
chemotherapy	6 (20)
EBRT and chemotherapy	5 (16.7)
anti-hormonal therapy	2 (6.6)
Recurrence after IORT	15 (50)
DFI (disease-free interval) month IORT to recurrence mean (range)	13 (3–60)
Type of relapse after IORT	
local	8 (26.7)
lymphnode	3 (10)
abdominal wall	4 (13.3)
distant	2 (6.7)
Therapy of relapse after IORT	10 (33.3)
chemotherapy	7 (23.3)
tumorresection	1 (3.3)
immuntherapy	1 (3.3)
IORT	1 (3.3)

**Table 3:** Complications during surgery and after IORT.

Complications: Clavien-Dindo	*n* (%)
I	3 (10)
II	8 (26.7)
III	14 (46.7)
IV	1 (3.3)
IORT Toxicity	
gastrointestinal obstruction	5 (16.6)
abscess	4 (13.3)
hematoma	1 (3.3)
neurologic peripheral	3 (10)
deep vein thrombosis	2 (6.6)
ureteral stenosis	5 (16.6)
ureterovaginal fistula	1 (3.3)

**Table 4:** Risk factors for complications.

Risk Factors	*p Value*
menopausal status	1
BMI	0.16
age at surgery	0.9
pre-surgical pelvic radiation	**0.02**
chemotherapy	0.58
bowel resection	0.07
urological surgery	0.36
lymphadenectomy	0.69
blood loss	0.85
surgery time	0.22
site of radiation (IORT)	0.16
mean radiation time	0.62
mean radiation doses in Gray	0.33

**Table 5:** Analysis of parameters that could have an impact on recurrence and survival.

					DSS			OS	
Variable	HR	95% CI	*p*	HR	95% CI	*p*	HR	95% CI	*p*
Type of cancer			**<0.001**			**0.008**			**0.02**
1 Cervix	1			1			1		
2 Endometrial cancer	0.42	0.05–3.33		0	0		0.47	0.06–3.73	
3 Uterine Sarcom	2.84	0.88–9.22		2.15	0.61–7.64		1.66	0.50–5.53	
4 Carcinosarcom of the ovary	37.7	2.23–637.3		15.96	1.37–185.9		15.4	1.36–176.65	
Hystological Type									
1 Squamous	1		0.08	1		0.06	1		0.8
2 Adenocarcinom + Clear cell	0.25	0.05–1.29		0.54	0.10–2.78		0.56	0.14–2.17	
3 Carsinosarcom	1.6	0.50–5.10		0.87	0.17–4.52		0.61	0.13–2.93	
4 Sarcom	3.48	0.38–31.75		5.46	0.9–29.91		4.53	0.88–23.38	
ECOG at time of IORT 0–1	2.47	0.85–7.14	0.09	5.5	1.63–19.23	**0.002**	3.41	1.18–9.80	**0.02**
Pre-IORT treatement	1.28	0.16 –9.90	0.82	0.51	0.11 –2.38	0.39	0.41	0.11–1.47	0.16
FIGO I-II versus FIGO III-IV	0.49	0.11–2.24	0.35	0.4	0.09–1.88	0.23	0.52	0.12–2.32	0.38
Number of applicators ≥ 10	1.19	0.40–3.49	0.76	1.04	0.27–3.93	0.96	1.11	0.35–3.55	0.86
Clean surgical margins	0.8	0.26–2.53	0.71	0.8	0.21–3.03	0.75	1.2	0.40–3.57	0.75
Complications > Clavien-Dindo Grade III	0.85	0.31–2.34	0.75	1.23	0.37–4.05	0.73	1.35	0.47–3.90	0.58

Legend RFS = relapse-free survival, DSS = disease-specific survival, OS = overall survival.

## Data Availability

The data presented in this study are available from the first author upon request.

## Abbreviations

The following abbreviations are used in this manuscript:

EBRT	External beam radiotherapy
DSS	Disease-specific survival
IORT	Intraoperative radiation therapy
RFS	Relapse-free survival
OS	Overall survival
